# Pristine MOF Materials for Separator Application in Lithium–Sulfur Battery

**DOI:** 10.1002/advs.202404834

**Published:** 2024-06-18

**Authors:** Zhibin Cheng, Jie Lian, Jindan Zhang, Shengchang Xiang, Banglin Chen, Zhangjing Zhang

**Affiliations:** ^1^ Fujian Key Laboratory of Polymer Materials College of Materials Science and Engineering Fujian Normal University Fuzhou 350007 China; ^2^ State Key Laboratory of Structural Chemistry Fujian Institute of Research on the Structure of Matter Chinese Academy of Sciences Fuzhou 350002 China

**Keywords:** energy storage, lithium sulfur batteries, MOF, separator, shuttle effect

## Abstract

Lithium–sulfur (Li–S) batteries have attracted significant attention in the realm of electronic energy storage and conversion owing to their remarkable theoretical energy density and cost‐effectiveness. However, Li–S batteries continue to face significant challenges, primarily the severe polysulfides shuttle effect and sluggish sulfur redox kinetics, which are inherent obstacles to their practical application. Metal‐organic frameworks (MOFs), known for their porous structure, high adsorption capacity, structural flexibility, and easy synthesis, have emerged as ideal materials for separator modification. Efficient polysulfides interception/conversion ability and rapid lithium‐ion conduction enabled by MOFs modified layers are demonstrated in Li–S batteries. In this perspective, the objective is to present an overview of recent advancements in utilizing pristine MOF materials as modification layers for separators in Li–S batteries. The mechanisms behind the enhanced electrochemical performance resulting from each design strategy are explained. The viewpoints and crucial challenges requiring resolution are also concluded for pristine MOFs separator in Li–S batteries. Moreover, some promising materials and concepts based on MOFs are proposed to enhance electrochemical performance and investigate polysulfides adsorption/conversion mechanisms. These efforts are expected to contribute to the future advancement of MOFs in advanced Li–S batteries.

## Graphical abstract

Metal‐organic frameworks are one type of promising porous materials to confine polysulfides within the cathode region. They can serve as effective separator modification layers to improve sulfur utilization, facilitate lithium‐ion transport, and suppress the shuttle effect of polysulfides, which holds great promises for the development of advanced lithium–sulfur batteries with high energy density and long cycle life.

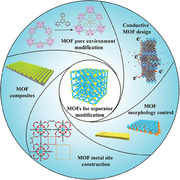

## 1 Introduction

In recent years, the rapid advancements in modern agriculture and industry have underscored the growing demand for renewable and clean energy.^[^ ^1^, ^2^, ^3^ ^]^ This demand has been further fueled by the increasing popularity of electric vehicles and mobile devices, resulting in a growing demand for innovative energy storage technologies. The electric vehicle industry, in particular, has set higher standards for power batteries, requiring them to have extended cycle life, higher energy density, and economical performance.^[^ ^4^, ^5^ ^]^ Currently, traditional lithium‐ion (Li^+^) batteries have reached a bottleneck with an energy density that is difficult to exceed 300 Wh kg^−1^.^[^ ^6^, ^7^, ^8^ ^]^ Hence, expediting the development of high‐energy‐density battery systems based on novel reaction mechanisms is crucial.^[^ ^9^, ^10^, ^11^ ^]^

Lithium–sulfur (Li–S) batteries are widely acknowledged as one of the most promising next‐generation electrochemical energy storage systems, characterized by a high theoretical specific capacity of 1675 mAh g^−1^ and energy density of 2600 Wh kg^−1^.^[^ ^12^, ^13^, ^14^ ^]^ Sulfur, as the primary cathode active material for Li–S batteries, has the advantages of being environmentally friendly, resourceful, and cost‐effective. In theory, Li–S batteries can achieve exceptional electrochemical performance through the redox reaction: 16Li + S_8_ → 8Li_2_S.^[^ ^15^, ^16^ ^]^ Specifically, the sulfur within the cathode undergoes reduction to lithium polysulfide during discharge, whereas the charging process oxidizes lithium polysulfide back to sulfur.^[^ ^17^, ^18^, ^19^, ^20^ ^]^ Despite the seemingly simple reaction, but the actual process is intricate. The intermediate products (Li_2_Sx, 4 ≤ x ≤ 8) formed in the charge/discharge process exhibit high solubility in liquid electrolytes. The soluble intermediate products have the ability to migrate across the commercial separator, traveling from the cathode to the anode, and interact with the Li metal. This reaction leads to the formation/accumulation of insoluble Li_2_S and Li_2_S_2_ on the surface of the Li anode, resulting in side reactions within the battery. This phenomenon, commonly referred to as the “shuttle effect”, has detrimental effects on Li–S batteries.^[^ ^21^, ^22^, ^23^ ^]^ The migration of polysulfides not only results in the irreversible depletion of active materials but also elevates the interfacial resistance between the cathode and electrolyte, leading to diminished coulombic efficiency, severe capacity degradation, and shortened cycle life of Li–S batteries.^[^ ^24^, ^25^, ^26^ ^]^

To accomplish high performance Li–S batteries, significant efforts have been dedicated to addressing the issue of polysulfides shuttling.^[^ ^27^, ^28^, ^29^, ^30^, ^31^ ^]^ These efforts predominantly revolve around innovations in cathode design and separator modification. Simply, most of protections of cathode are using functional materials to encapsulated sulfur.^[^ ^32^, ^33^, ^34^, ^35^, ^36^ ^]^ However, during the period of charging and discharging cycle, the cathode material will be unstable by degrees and cannot restrict the shuttling of polysulfides. Moreover, excessive incorporation of inactive materials into the cathode can reduce the overall energy density.^[^ ^37^, ^38^, ^39^ ^]^ It is widely acknowledged that addressing the challenges in Li–S batteries necessitates attention beyond the sulfur cathode alone. A promising approach is to advance the development of functional separators to improve the overall electrochemical performance of Li–S batteries.^[^ ^40^, ^41^, ^42^, ^43^ ^]^ The conventional separators are typically composed of polymer porous membranes featuring large macropores. These macropores facilitate the unrestricted migration of soluble polysulfides through the separators, leading to unavoidable performance degradation during charging and discharging cycles.^[^ ^44^, ^45^, ^46^ ^]^ Inspired by the pioneering work of Manthiram,^[^ ^47^ ^]^ who introduced bifunctional microporous carbon paper as interlayer to enhance sulfur utilization, various materials ranging from carbon materials to metal oxides and polymers have been explored for separator modification in Li–S batteries.^[^ ^48^, ^49^, ^50^ ^]^ Numerous reviews have summarized the positive impact of these materials on rate performance and cycle life improvement. While physical confinement and electrostatic repulsion offered by modified layers have shown promise in mitigating the shuttle effect of polysulfides, challenges persist due to limited surface area and fewer active sites, hindering the achievement of satisfactory cycling stability.^[^ ^51^, ^52^, ^53^ ^]^ Additionally, the predominantly monofunctional nature of these materials remains a significant impediment to effectively restraining the polysulfides shuttling and enhancing reaction kinetic within the cell.^[^ ^54^, ^55^, ^56^ ^]^

**Figure 1:**
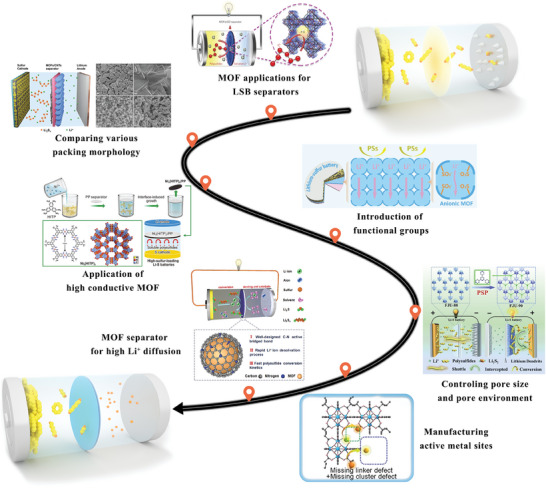
Development of design strategies of MOF‐based separators for optimizing Li–S battery performance.

In recent years, metal‐organic frameworks (MOFs) have attracted significant interest among researchers owing to their highly ordered structure, tunable pore sizes, and rich porosity.^[^ ^57^, ^58^, ^59^, ^60^ ^]^ Notably, these distinctive features of MOFs align with the goal of reducing the polysulfides shuttling.^[^ ^61^, ^62^, ^63^ ^]^ The well‐balanced pore sizes distribution and the substantial specific surface area of MOFs serve as effective barriers, physically impeding the undesired shuttle effect.^[^ ^64^, ^65^ ^]^ The metals embedded in MOFs, such as Ni and Co, exhibit remarkable catalytic activity, acting as Lewis acids that interact with soluble polysulfides.^[^ ^66^, ^67^ ^]^ Moreover, some unique functional groups present in MOFs, such as ‐SO_3_H and ‐NH_2_, play a crucial role in restricting the migration of polysulfides through electrostatic interactions.^[^ ^68^, ^69^ ^]^ Consequently, an increasing number of researchers have presented compelling evidence to demonstrate the excellent performance of MOFs when integrated into Li–S battery separators (**Figure** **1**).^[^ ^70^, ^71^, ^72^ ^]^

**Figure 2:**
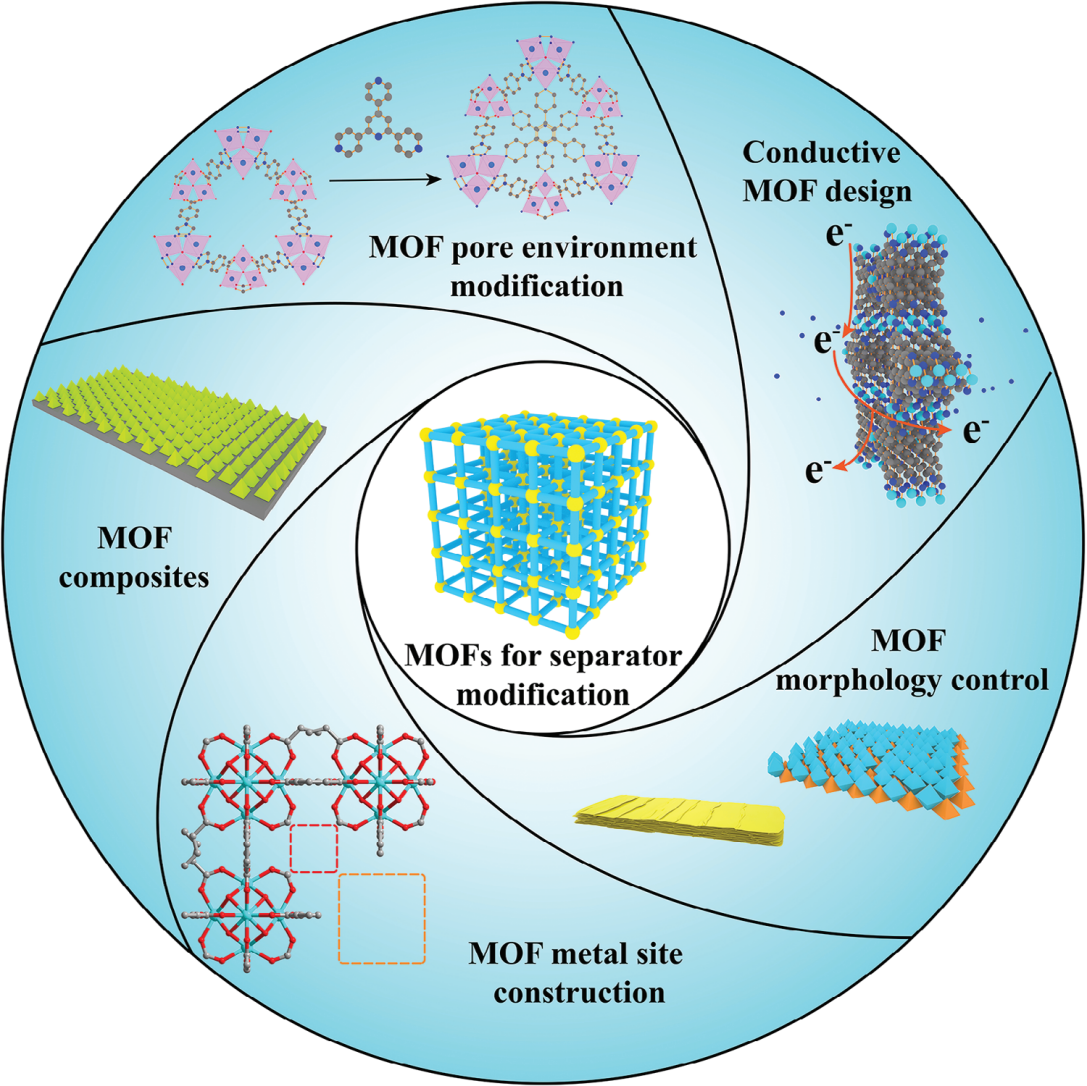
Various design strategies of MOFs in Li–S battery separator.

Nevertheless, previous reviews on MOF applications in Li–S batteries mainly focused on employing MOFs for modifying the sulfur cathode or using MOF derivatives to modify separators. This review endeavors to present a fresh perspective by providing a comprehensive overview of recent progress in utilizing pristine MOFs to modify separators in Li–S batteries. Encompassing various aspects, the review covers MOF pore environment modifications, MOF metal site constructions, conductive MOF designs, MOF morphology control, and MOF composites (**Figure** **2**), delving into their latest progress and current limitations. Furthermore, the review offers a comprehensive discussion of the working mechanisms involved in the application of these multifunctional composite separators in Li–S batteries. Finally, it concludes with an outlook that not only outlines potential solutions for addressing current challenges but also suggests research directions for the near future.

## 2 Design Strategies for MOFs Applied to Li–S Battery Separator

### 2.1 MOF Pore Environment Modification

Structural design flexibility is one of the most notable advantages of MOF materials over other porous materials.^[^ ^73^, ^74^ ^]^ This remarkable attribute enables the modification of existing MOF materials to incorporate adjustable pore environments, including pore size, pore charge, and more. Importantly, these characteristics can be readily and efficiently customized at a later stage, allowing for precise control of material properties tailored to specific applications.^[^ ^75^ ^]^ When considering the use of MOFs as separator‐modifying materials, a promising approach involves regulating and optimizing the pore environment to restrict the migration of polysulfides.^[^ ^76^ ^]^ Simultaneously, it becomes feasible to introduce active sites that enhance host‐guest interactions.^[^ ^77^, ^78^ ^]^ This exceptional structural adaptability holds significant promise for harnessing MOF materials to improve the performance of Li–S battery separators.

**Figure 3:**
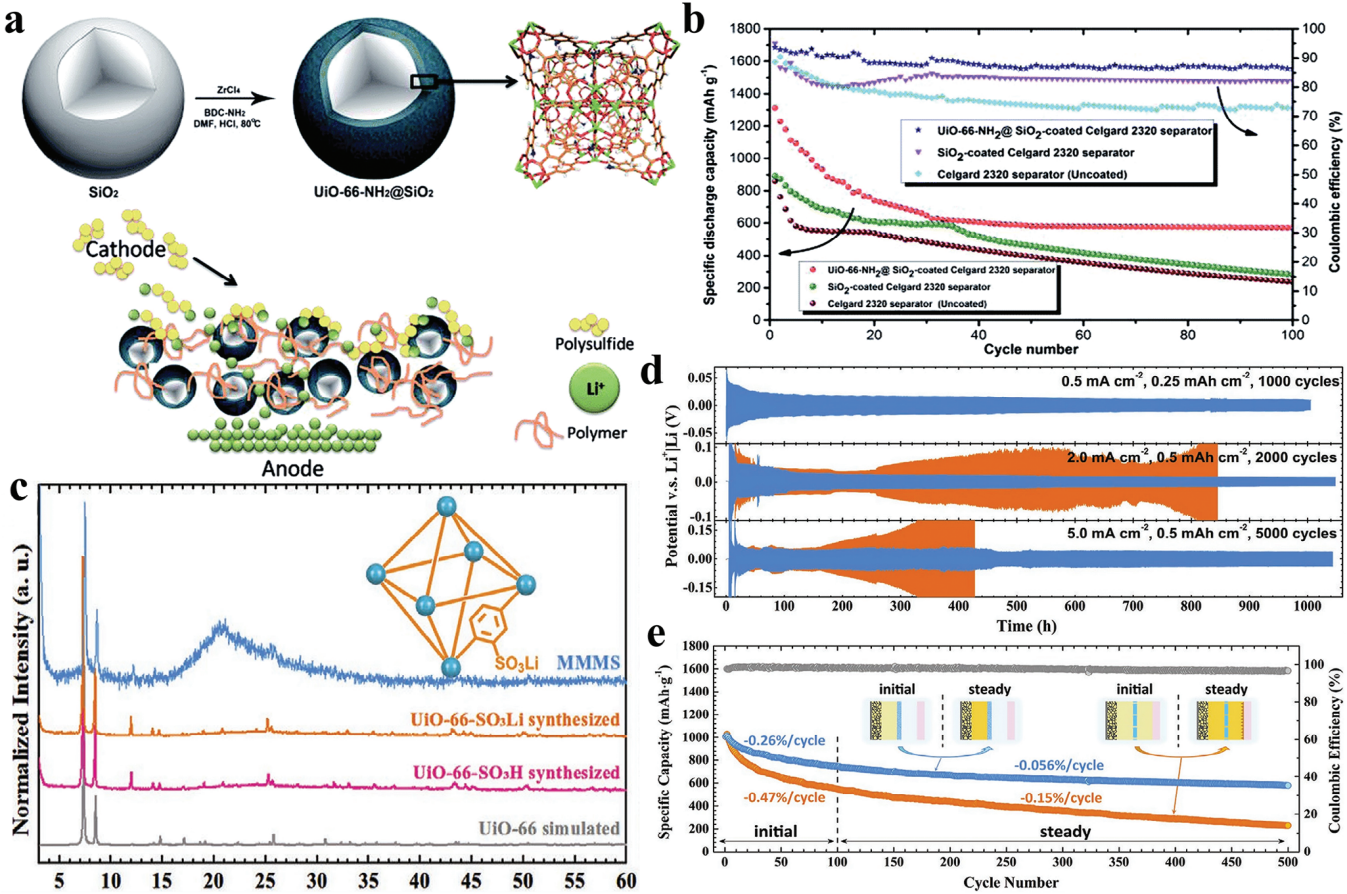
a) Synthesis of the UiO‐66‐NH_2_@SiO_2_ and the proposed membrane action mechanism. b) Cycling performance. Reproduced with permission.^[^ ^79^ ^]^ Copyright 2018, Royal Society of Chemistry. c) XRD spectra of UiO‐66, UiO‐66‐SO_3_H, UiO‐66‐SO_3_Li, and MMMS. d) Lithium plating/stripping performance comparison between the Li|PP|Li and Li|MMMS|Li configurations under various current rates. e) Long cycling performance at 0.5 C. Reproduced with permission.^[^ ^81^ ^]^ Copyright 2020, Wiley‐VCH.

Initially, researchers began tailoring MOF functionality through linker modifications, involving the incorporation of specific functional groups into the MOF's organic framework. Suriyakumar et al. fabricated amine‐functionalized UiO‐66 (UiO‐66‐NH_2_) for separator modification (**Figure** **3**a).^[^ ^79^ ^]^ The resulting amine‐functionalized MOF can interact with diffusing polysulfides through hydrogen bonding, effectively restraining the migration of polysulfides toward the anode. As illustrated in Figure 3b, the cell with a modified separator exhibited the high initial discharge capacity of 1400 mAh g^−1^ at 0.1, corresponding to 83.5% of the theoretical capacity of sulfur. Even after 100 cycles, the capacity remained at ≈600 mAh g^−1^, showing good cycling performance (**Table** **1**). In addition to the shuttle effect, the issue of dendrites in the Li anode also affects the cycling stability of Li–S batteries.^[^ ^80^ ^]^ In another study, Wang et al. prepared UiO‐66‐SO_3_Li through cationic exchange from UiO‐66‐SO_3_H and incorporated it with poly(vinylidene fluoride) (PVDF) using a mixed‐matrix membrane (MMM) method.^[^ ^81^ ^]^ The composite membrane adorned with plentiful sulfonate anionic groups (‐SO_3_^−^) showed XRD diffraction patterns consistent with the simulated results (Figure 3c). This membrane displayed both an electrostatic repulsion effect on polysulfides and well‐defined conductive channels to efficiently regulate Li^+^ flux. Consequently, the Li–S cell utilizing the MMM exhibited remarkable stability in Li plating/stripping, enduring over 1000 h at 5 mA cm^−2^ (Figure 3d). Furthermore, it sustained 500 cycles at 0.5 C with a low average capacity decay rate per cycle of 0.056% (Figure 3e). These studies offer an excellent platform to explore the correlation between the MOF pore environment and its functional for limiting polysulfides shuttling and suppressing lithium dendrite growth.

**Table 1:** MOF‐modified separators for Li–S batteries.

Modified material	Sulfur in composite [wt.%]	Sulfur loading [mg cm^−2^]	Areal mass loading [mg cm^−2^]	Initial discharge capacity [mAh g^−1^]	Cycling performance [mAh g^−1^]	Rate performance [mAh g^−1^]	References
**MOF pore environment**							
UiO‐66‐NH_2_@SiO_2_	/	0.5	/	1400 (0.1 C)	600 (100th, 0.1 C)	/	[79]
UiO‐66‐SO_3_Li	/	2.0	/	1020 (0.5 C)	580 (500th, 0.5 C)	552 (5 C)	[81]
Ms‐9.0‐NSP	70	2.4	/	1316 (0.5 C)	1000 (1000th, 1 C)	963 (2 C)	[84]
FJU‐88	66	1	1.27	955.7 (0.5 C)	493.8 (500th, 1 C)	596 (5 C)	[85]
FJU‐90	66	1	1.27	1104.4 (0.5 C)	826.5 (500th, 1 C)	827.2 (5 C)	[85]
Li‐MOF/RGO	80	1.2‐1.4	0.25	956 (0.5 C)	/	742 (2 C)	[86]
**MOF open metal site construction**							
CSUST‐1	70	2‐5	0.3	1468 (0.1 C)	538 (1200th, 2 C)	541 (5 C)	[95]
B/2D MOF‐Co	80	1.5	1.25	1112 (0.1 C)	703 (200th, 0.5 C)	478 (5 C)	[96]
Zn/Co‐ZIF	/	2.1	0.1	1304 (0.5 C)	630 (1000th, 2 C)	788 (3 C)	[97]
D‐UiO‐66‐NH2‐4/G	70	1.5	0.154	/	756 (700th, 3 C)	840 (3 C)	[98]
aMIL‐88B	/	4.3	/	/	740 (500th, 1 C)	610 (5 C)	[99]
UiO‐66D2	75	2	/	1063 (0.2 C)	785 (500th, 1 C)	/	[100]
**Conductive MOF design**							
Ni_3_(HITP)_2_	69.1	0.8‐1.2	0.066	1404.6 (0.1 C)	500.7 (700th, 0.5 C)	1046.8 (0.1 C)	[104]
Ni_3_(HITP)_2_	/	/	0.33	1220.1 (0.1 C)	585.4 (300th, 0.5 C)	800.2 (2 C)	[105]
Ni‐HAB@CNT	/	2.5	/	1015 (0.1 C)	1070 (200th, 0.2 C)	799 (3 C)	[106]
**MOF morphology control**							
Y‐FTZB	63	1	/	1101 (0.25 C)	557 (300th, 0.25 C)	/	[111]
HKUST‐1	63	1	/	1032 (0.25 C)	197 (300th, 0.25 C)	/	[111]
ZIF‐7	63	1	/	1025 (0.25 C)	452 (300th, 0.25 C)	/	[111]
ZIF‐8	63	1	/	989 (0.25 C)	403 (300th, 0.25 C)	/	[111]
UiO‐66	80	2.5‐3	/	1147.4 (0.2 C)	964.1 (200th, 0.5 C)	955.8 (2 C)	[112]
2D‐Cu‐BDC	70	1.8‐2.3	0.12	1231 (0.1 C)	603 (500th, 1 C)	565 (3 C)	[113]
In/Zr‐BTB	75	/	0.011	840.7 (2 C)	679.5 (500th, 2 C)	637.77 (5 C)	[114]
2D‐NiCo MOF/CNT	/	1.2	0.08‐0.12	1132.7 (0.5 C)	709.1 (300th, 1 C)	691.7 (3 C)	[115]
**MOF composites**							
HKUST‐1@GO	56	0.6‐0.8	/	1126 (0.5 C)	870 (100th, 1 C)	488 (3 C)	[121]
Zn(II)‐based MOF@GO	70	0.6‐0.8	/	1118 (1 C)	657 (1000th, 1 C)	/	[122]
HAR‐ZIF‐67/G	50	3.5	0.39	882.1 (1 C)	831 (500th, 1 C)	400 (3.2 C)	[123]
CNT@ZIF‐30/PP	80	1.2	0.9	1588.4 (0.2 C)	870.3 (100th, 0.2 C)	583.2 (2 C)	[124]
MOF@CC	/	0.422	0.422	1063(0.5 C)	920 (100th, 0.5 C)	765 (5 C)	[125]
**MOF polymer composites**							
MOF@PVDF‐HFP	87	1‐1.5	/	1231 (0.1 C)	936 (200th, 2 C)	633 (3 C)	[128]
HPP‐20	69.1	0.8‐1.2	/	1163.7 (0.5 C)	500.7 (700th,0.5 C)	1046.8 (0.1 C)	[129]
Triple‐layer	/	2.4‐3	/	1365.0 (0.5 C)	1365 (700th,0.5 C)	766.6 (2 C)	[130]
Z‐PMIA	/	/	/	1391.2 (0.2 C)	961 (350th,0.2 C)	774.8 (2 C)	[133]

**Figure 4:**
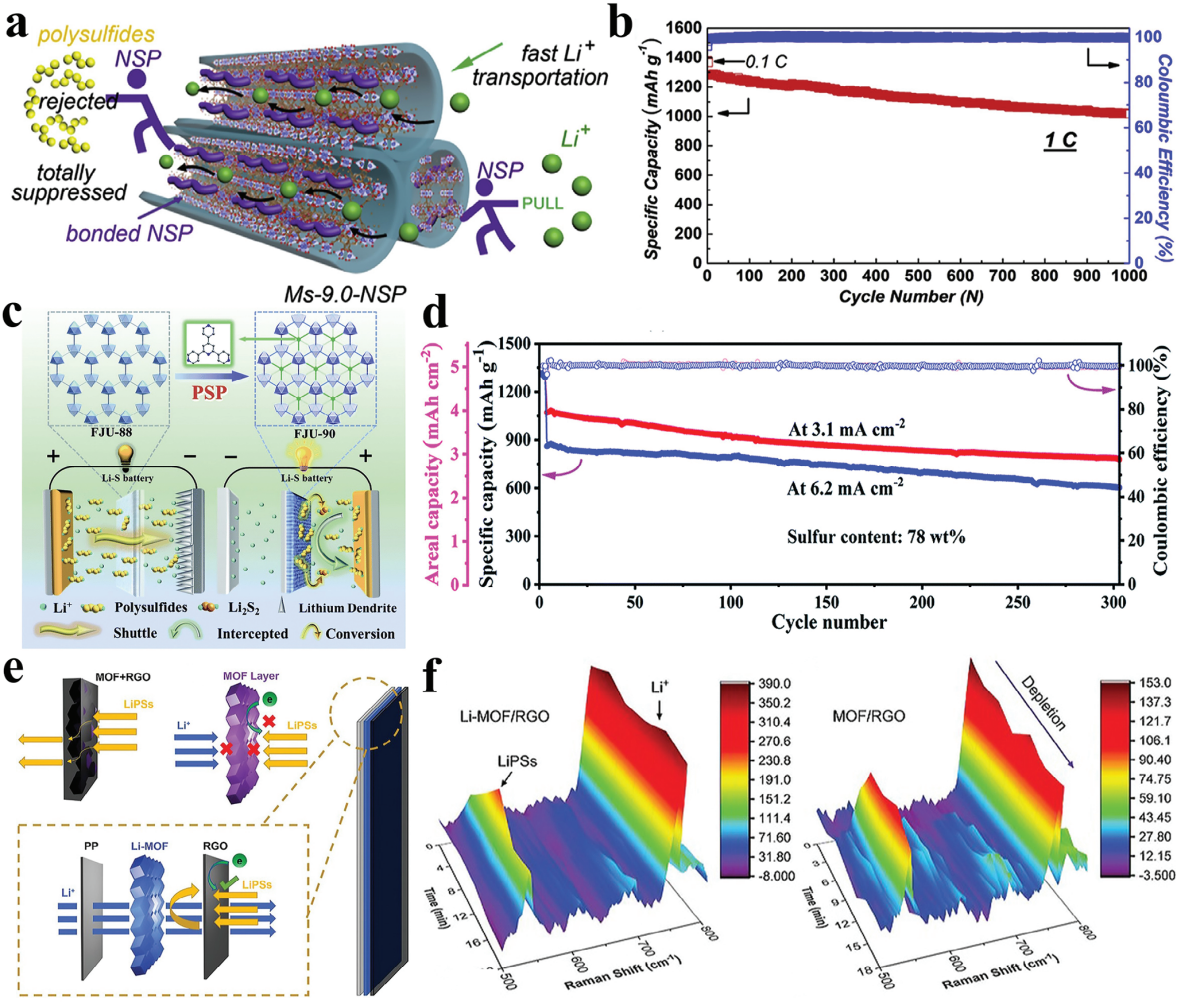
a) Schematic illustration for Ms‐9.0‐NSP diffusion of both polysulfides and Li^+^. b) Long cycling performance at 1 C. Reproduced with permission.^[^ ^84^ ^]^ Copyright 2020, Elsevier. c) Schematic comparison of the separator with FJU‐90 and FJU‐88. d) Cycling performances of the FJU‐90/PP cell. Reproduced with permission.^[^ ^85^ ^]^ Copyright 2021, Royal Society of Chemistry. e) Schematic illustrating the mass and charge transfer mechanisms across various functional separators. f) In situ Raman spectra. Reproduced with permission.^[^ ^86^ ^]^ Copyright 2021, Wiley‐VCH.

Building upon earlier investigations into mitigating the polysulfides shuttling and suppressing the growth of Li dendrites, separators with functional of “ion sieve” have been developed. These separators are specifically designed to impede the shuttle effect while promoting the rapid transport and uniform deposition of Li^+^.^[^ ^82^, ^83^ ^]^ Chang et al. conducted experiments to assess the impact of MOF pore size on polysulfide trapping and Li^+^ transport, comparing three pore sizes.^[^ ^84^ ^]^ They observed that Ms‐9.0, with its larger pore size, exhibited higher Li^+^ transport capacity and lower polarization. However, it was observed that the copper sites within the Ms‐9.0 pore exhibited excessive strength for polysulfides adsorption, leading to substantial initial sulfur loss. To address this issue, they incorporated a negative charged sulfonic polymer (NSP) into Ms‐9.0, creating Ms‐9.0‐NSP. With the NSP binding to the bare copper sites within the Ms‐9.0‐NSP channel, the problematic interaction between copper sites and polysulfides (metal‐S2‐x) was greatly reduced. As shown in **Figure** **4**a, after introducing NSP, the pore size of Ms‐9.0‐NSP was reduced to 6.9 Å and the charge environment within the pore channel is altered. The narrow channels constructed by the negatively charged NSP prevented the entrapment or diffusion of negatively charged polysulfides, while simultaneously attracting positively charged Li^+^ ions, expediting their transport and consequently reducing voltage polarization. When cycled at 1 C, the Ms‐9.0‐NSP cell delivered an initial discharge capacity of 1279 mAh g^−1^, corresponding to 76.3% of the theoretical capacity of sulfur, and retained 1017 mAh g^−1^ after 1000 cycles, demonstrating outstanding cycling performance for the Ms‐9.0‐NSP cell (Figure 4b). The incorporation of NSP effectively modulates the charge environment and pore size of MOF, enabling efficient Li^+^ transport and polysulfides interception. In order to accurately control the pore structure of modified layer, Cheng et al. implemented a unique Pore‐space‐partitioned strategy to modify MOF framework pore chemistry.^[^ ^85^ ^]^ As presented in Figure 4c. They developed FJU‐90 by incorporating the nitrogen‐rich ligand 2,4,6‐tris(4‐pyridyl)pyridine into FJU‐88, effectively optimizing the pore structure and reducing pore size. This precisely customized MOF features a well‐balanced pore size, a generous specific surface area, and an abundance of catalytic sites. These properties work synergistically to suppress polysulfides shuttling, enhance Li^+^ ion conduction and facilitate catalytic polysulfide conversion. The outcome is remarkable cycling stability and rate performance, even in high sulfur content cathodes (Figure 4d). Remarkably, the achieved electrochemical performance for FJU‐90/PP matches or surpasses that of previously reported cells utilizing MOF separators (Table 1). In addition to homogenizing the flux of Li^+^ through pore‐space‐partition, the pre‐introduction of Li^+^ into the MOFs can also efficiently facilitate the Li^+^ transport. Zhou et al. employed lithium bis(trifluoromethylsulfonyl)imide (LiTFSI) to directly modify ZIF‐67, resulting in a Li^+^ inserted ZIF‐67 (Li‐MOF) (Figure 4e).^[^ ^86^ ^]^ In situ Raman spectroscopy revealed that the formed Li‐MOF functional layer effectively hindered the shuttling of polysulfides, thereby increasing the utilization of the sulfur species (Figure 4f). The Li‐ions inserted into the pores served as a conductive pathway for Li^+^ transport, promoting ion transfer kinetics and facilitating mass transfer across the layers. A battery assembled with Li‐MOF/RGO separator exhibited a minimal fading rate of 0.089% over 600 cycles at a 1 C current, demonstrating excellent cycling performance.

### 2.2 MOF Metal Site Construction

The structural design approach for MOFs can also be extended to the arrangement of metal sites within the structure.^[^ ^87^ ^]^ Metal site play a crucial role in catalyzing the cell's redox reactions, expediting reaction kinetics, and offering active sites for polysulfide adsorption.^[^ ^88^, ^89^, ^90^ ^]^ Recent developments have highlighted the significance of defect engineering in modulating the catalytic performance and electronic structure of electrocatalysts.^[^ ^91^, ^92^, ^93^ ^]^ The introduction of defects, such as metal vacancies, or the incorporation of other metals into the MOF to create synergistic effects, increases the chemical interaction between MOFs and polysulfides.^[^ ^94^ ^]^ This, in turn, enables a more effective utilization of metal‐catalyzed active sites to significantly accelerate the kinetics of polysulfide conversion reactions at various stages.

**Figure 5:**
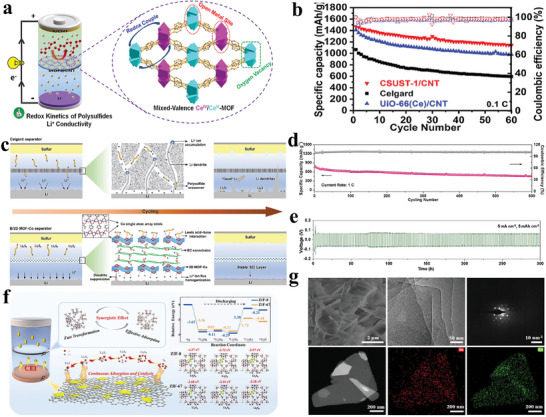
a) Diagram depicting the framework structure of CSUST‐1. b) Cycling performance of the different cells at 0.1 C. Reproduced with permission.^[^ ^95^ ^]^ Copyright 2021, American Chemical Society. c) Schematic representation of Li–S batteries featuring Celgard and B/2D MOF‐Co separators. d) Long cycling performance of the B/2D MOF‐Co cell at 1 C. e) Stability of Li stripping and plating in Li//Li symmetric cells utilizing a B/2D MOF‐Co separator. Reproduced with permission.^[^ ^96^ ^]^ Copyright 2020, Wiley‐VCH. f) Reaction mechanism and density functional theory calculation of Zn/Co‐ZIF nanosheets on polysulfides. g) The morphological analysis of Zn/Co‐ZIF nanosheets. Reproduced with permission.^[^ ^97^ ^]^ Copyright 2022, Elsevier.

Jin et al. fabricated a mixed‐valence Ce‐MOF (CSUST‐1) with isolated Ce (IV, III) arrays (**Figure** **5**a).^[^ ^95^ ^]^ The CSUST‐1/CNT composite exhibited effective adsorption, accelerated polysulfides redox kinetic conversion, and improved Li^+^ transport attributed to the isolated Ce (IV, III) arrays, abundant oxygen vacancies, and accessible metal sites. The CV curves of the symmetrical cells indicated that the high redox rate in cell was attributed to the exposed metal sites in CSUST‐1. The corresponding battery exhibited outstanding performance, delivering an initial areal capacity of 8.7 mAh cm^−2^ at 0.1 C, and maintaining an areal capacity of 6.1 mAh cm^−2^ after 60 cycles even at 8 mg cm^−2^ areal sulfur loading (Figure 5b). This work clarified the mechanism about mixed‐valence metal electrocatalyst for polysulfides conversion. Guo et al. designed a dual‐function separator by layering ultrathin MOF nanosheets with bacterial cellulose in a layer‐by‐layer assembly (Figure 5c).^[^ ^96^ ^]^ These ultrathin MOF‐Co nanosheets exposed regularly arranged cobalt and oxygen atoms on their surfaces. At the anode side, the presence of O atoms results in the homogenization of Li‐ion flux by their adsorption onto Li^+^, thus ensuring uniform Li stripping/plating. At the cathode side, Co atoms effectively mitigate the shuttle effect by capturing polysulfides through Lewis acid‐base interactions. Consequently, the Li–S cell equipped with this bifunctional B/2D MOF‐Co membrane exhibits exceptional long‐cycle stability (Figure 5d) and high Li stripping and plating stability at 5 mA cm^−2^ (Figure 5e). Bimetallic MOF materials have also been investigated for enhancing separator performance through modification. Zhou et al. conducted a comparative study of different metal centers within MOFs regarding their adsorption and catalytic capabilities for polysulfides, using a combination of DFT calculations and controlled experiments (Figure 5f).^[^ ^97^ ^]^ Both theoretical calculations and experimental findings concurred that Zn and Co metal centers demonstrated superior adsorption and catalytic properties for polysulfides, respectively. Corresponding SEM, TEM, SAED pattern, and TEM elemental mapping images reveal that Zn/Co‐ZIF has a nanosheet structure with a uniform distribution of Zn and Co (Figure 5g). By regulating the metal center sites within the bimetallic nanosheet, the kinetics of polysulfide conversion can be significantly improved.

**Figure 6:**
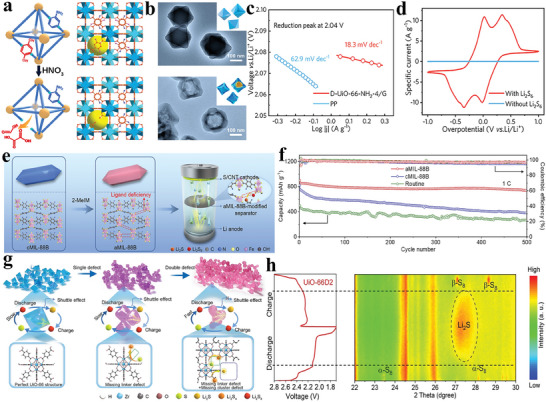
a) Schematic illustration the frameworks structure of D‐UiO‐66‐NH_2_. b) TEM images of UiO‐66‐(OH)_2_‐NH_2_‐4 and D‐UiO‐66‐NH_2_‐4. c) Tafel plot about polysulfides reduction. d) CV curves of D‐UiO‐66‐NH_2_‐4/G symmetric cell. Reproduced with permission.^[^ ^98^ ^]^ Copyright 2021, American Chemical Society. e) Diagram illustrating the application of aMIL‐88B as a Li–S separator modified layer. f) Long cycling performances at 1 C. Reproduced with permission.^[^ ^99^ ^]^ Copyright 2021, Elsevier. g) Schematic illustration of UiO‐66P, UiO‐66D1, and UiO‐66D2. h) In situ XRD of UiO‐66D2 cell. Reproduced with permission.^[^ ^100^ ^]^ Copyright 2023, Wiley‐VCH.

Li et al. directionally engrave D‐UiO‐66‐NH_2_ through a structural defect engineering based on targeted nitrolysis (**Figure** **6**a).^[^ ^98^ ^]^ The TEM (Figure 6b) image shows that D‐UIO‐66‐NH_2_ exposes defects. DFT calculations and XPS analysis confirmed that the increased number of backbone defects in D‐UiO‐66‐NH_2_ attenuated the Li–S bonding and enhanced electron transfer to and from polysulfides. The Tafel plots (Figure 6c) and cyclic voltammetry (CV) (Figure 6d) curves also illustrated that this defect‐rich design improved the redox transformation of polysulfides with bidirectional catalytic activity. Zhang et al. utilized a ligand competition approach to prepare an amorphous MOF (aMIL‐88B) (Figure 6e).^[^ ^99^ ^]^ In contrast to the crystalline cMIL‐88B, the presence of ligand defects in the amorphous MOF matrix resulted in enhanced electrical conductivity, increased exposure of active sites, and more robust spatial and ligand interactions with polysulfides. These advantages facilitate polysulfide fixation and catalysis. Attributed to these characteristics, the Li–S cells employing an aMIL‐88B‐modified separator exhibit outstanding cycling stability, maintaining a capacity of 740 mAh g^−1^ after 500 cycles (Figure 6f and Table 1). Wang et al. introduced an innovative dual‐defect MOF to facilitate multi‐step catalysis in polysulfide redox reactions (Figure 6g).^[^ ^100^ ^]^ By integrating experimental findings with DFT calculations, they illustrated that UiO‐66, featuring two distinct defects, the linker defect and cluster defect significantly catalyze the reaction of the S_8_→Li_2_S_4_ and Li_2_S_4_→Li_2_S, respectively. The presence of linker defects in UiO‐66 renders the Zr active sites accessible, thereby enhancing the catalytic efficiency of converting sulfur to Li_2_S_4_. However, these defects exhibit limited effectiveness in converting Li_2_S_4_ to Li_2_S. Furthermore, the electronic structure of UiO‐66 undergoes alterations due to cluster defects, resulting in an increased affinity between active sites and short‐chain polysulfides. This heightened affinity facilitates the complete transformation of short‐chain polysulfides into Li_2_S. The proximity of these diverse defects allowed for a synergistic effect during sulfur reduction reactions, enhancing the overall catalytic efficiency for polysulfide conversion. The in situ XRD images of Li–S cell with UiO‐66D2 modified separator further corroborate the complete conversion of sulfur to Li_2_S during the discharge, and the subsequent full re‐conversion of Li_2_S to sulfur in the subsequent charging process (Figure 6h). As a result, even under ultrahigh areal sulfur loading of 12.9 mg cm^−2^, the cells equipped with defect‐rich UiO‐66‐modified separator retained a substantial area capacity of 10.4 mAh cm^−2^ for 45 cycles at 0.05 C. These works offer distinctive insights into designing MOF defects as functional sites for catalyzing polysulfides conversion.

### 2.3 Conductive MOF Design

MOFs can effectively restrain shuttle effects and enhance polysulfide conversion rates through rational design. However, it's worth noting that most MOFs exhibit poor electrical conductivity, which contributes to increased interfacial resistance from the separator to the cathode.^[^ ^101^ ^]^ In light of the growing number of design strategies for conductive MOFs in recent years, the utilization of highly conductive MOFs as functional modifiers has emerged as an effective approach.^[^ ^102^, ^103^ ^]^ Coating the conductive MOF layer on the commercial separator serves a dual purpose: it serves as a physical interception layer, inhibiting the migration of polysulfides toward the lithium anode, while simultaneously reducing the interfacial resistance generated by the MOF‐modified layer. Furthermore, it leads to an increase in the reaction rate during the conversion process.

**Figure 7:**
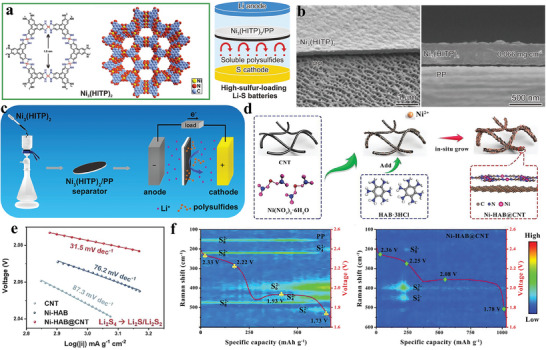
a) Diagrammatic representation of 2D Ni_3_(HITP)_2_ for separator modification. b) Cross sectional SEM images for Ni_3_(HITP)_2_/PP separator. Reproduced with permission.^[^ ^104^ ^]^ Copyright 2018, Wiley‐VCH. c) Diagram depicting the fabrication process of the Ni_3_(HITP)_2_/PP separator via filtration, and its integration into the Li–S Battery. Reproduced with permission.^[^ ^105^ ^]^ Copyright 2019, American Chemical Society. d) Schematic diagrams of the fabrication of Ni‐HAB@CNT. e) The tafel profiles and f) In situ time‐resolved Raman spectra obtained during the discharging. Reproduced with permission.^[^ ^106^ ^]^ Copyright 2023, Wiley‐VCH.

Zang et al. selected a multifunctional MOF Ni_3_(HITP)_2_ (HITP = 2,3,6,7,10,11‐hexaimi‐notriphenylene), which exhibits high conductivity, ordered microporous structure and large specific surface area (**Figure** **7**a).^[^ ^104^ ^]^ They applied this MOF directly to commercial PP separators using interface‐induced growth technology, resulting in an ultra‐thin and homogeneous modification with a low mass loading of 0.066 mg cm^−2^ (Figure 7b). Unlike traditional MOFs, this 2D layered MOF, formed by π‐conjugated ligands, possesses unique structural characteristics and stands out for its exceptional electrical conductivity. The conductivity of Ni_3_(HITP)_2_ reaches up to 4000 S m^−1^, surpassing that of activated carbon and porous graphite by fourfold. When incorporated into Li–S batteries as part of the Ni_3_(HITP)_2_/PP separator, it demonstrates remarkable cycle performance. The Ni_3_(HITP)_2_/PP separator exhibited an initial discharge capacity of 1244 mAh g^−1^, corresponding to 74.2% of the theoretical capacity of sulfur. Impressively, even after 100 cycles, it maintained a capacity of 1139 mAh g^−1^, retaining 92% of its initial capacity. Moreover, it displayed an average capacity attenuation rate of merely 0.08% per cycle. Despite the excellent electrochemical performance achieved by this method, regulating the separator modification layer formed through interface growth remains challenging. Consequently, researchers have also explored alternative, more straightforward techniques for preparing conductive MOF‐based separator modification layers. Chen et al. utilized a straightforward filtration method to create a modified separator using Ni_3_(HITP)_2_ (Figure 7c).^[^ ^105^ ^]^ Initially, Ni_3_(HITP)_2_ was synthesized through a solvothermal synthesis process, and subsequently, it was blended with NMP and PVDF dispersions. The resulting dispersed solution was then directly filtered onto a commercial PP separator. This uncomplicated and efficient approach yielded similarly enhanced performance for Li–S batteries. Guo et al. selected Ni^2+^ and the smallest π‐conjugated hexaaminobenzene as a linker to in situ assemble high crystallinity Ni‐HAB 2D conductive MOFs through dsp^2^ hybridization on the surface of CNTs (Figure 7d).^[^ ^106^ ^]^ The resulting Ni‐HAB@CNT material was employed as a modified separator layer for Li–S batteries. This unique π‐d conjugated Ni‐HAB 2D c‐MOF exhibited excellent conductivity, minimal steric hindrance, and a high density of delocalized electrons, thereby accelerating the redox kinetics of lithium polysulfides. Both the Tafel profiles, displaying an amplified cathodic peak within the range of 2.04–2.11 V (Figure 7e), and in situ time‐resolved Raman spectra (Figure 7f) effectively indicated rapid reaction kinetics in the Ni‐HAB@CNT/PP cells. Even with a low electro/sulfur ratio of 5 µL mg^−1^ and high areal sulfur loading of 6.5 mg cm^−2^, the Li–S cells equipped with a Ni‐HAB@CNT modified separator achieved a high areal capacity of 6.29 mAh cm^−2^.

### 2.4 MOF Morphology Control

In addition to the design of the pore channels at the microscopic scale, the macroscopic design of the MOF material modification layer in the application is also important.^[^ ^107^, ^108^, ^109^ ^]^ Most of MOFs are stacked in microparticles when they acted as separator functionalization material. However, the gap caused by this loose packing allows most polysulfides to migrate from the grain boundary to the anode rather than from the channel of MOFs, which goes against the original intention of using structure of MOF materials to suppress shuttle effect, so the morphology and packing morphology of MOF materials will affect the inhibition ability of the modified layer directly.^[^ ^110^ ^]^ Therefore, the regulation of morphology of MOFs through rational design can significantly improve the grain boundary caused by the accumulation of MOFs, compel polysulfides to transfer through the pores of MOFs to reduce the migration from grain boundary and enhance the functional modification effect of MOFs.

**Figure 8:**
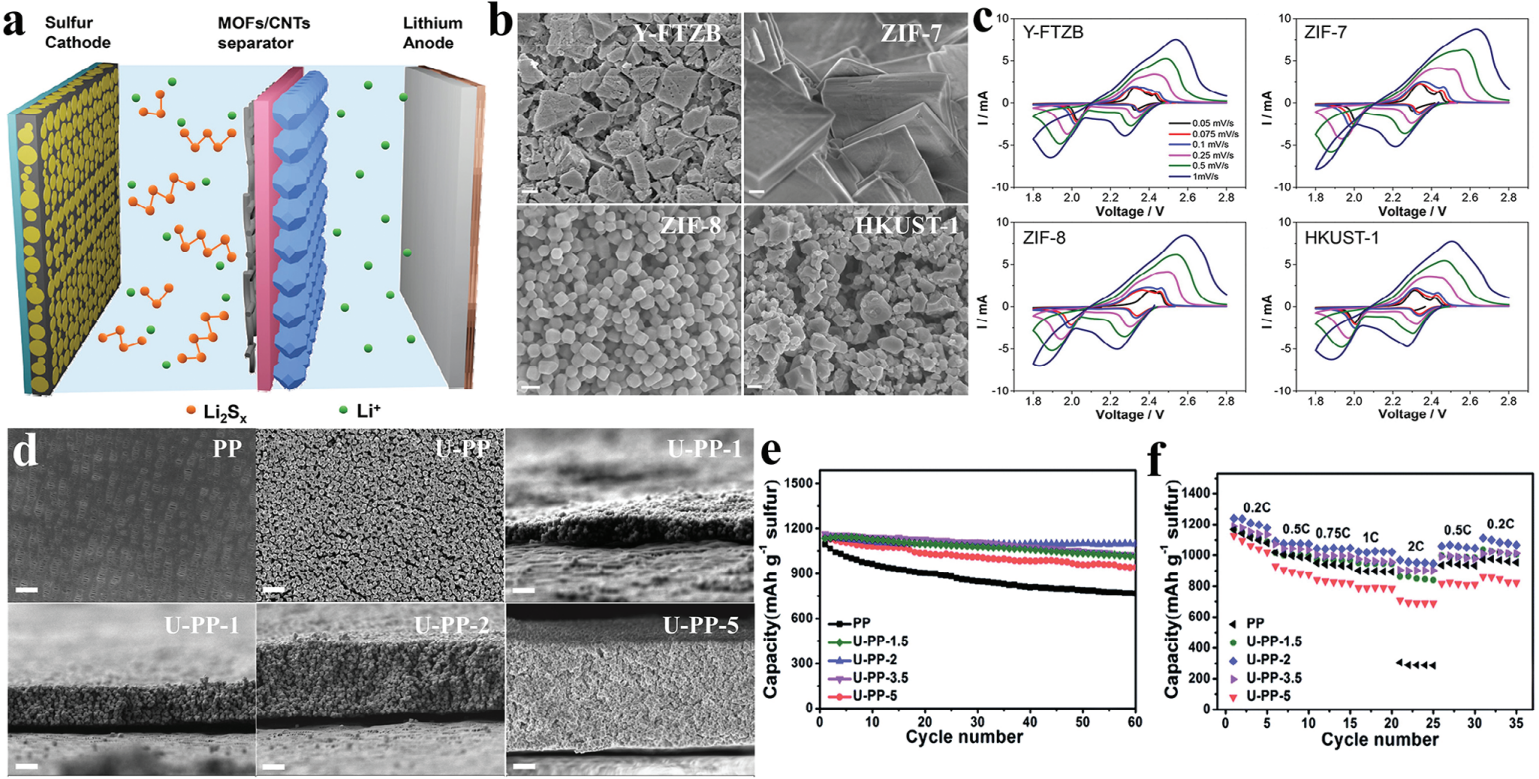
a) Diagram illustrating the configuration of the Li–S battery. b) SEM images of the stacking structure of the different MOF/PP separators. The scale bar is 200 nm. c) CV curves of the cells using MOF/PP separators. Reproduced with permission.^[^ ^111^ ^]^ Copyright 2017, American Chemical Society. d) SEM images of the PP, U‐PP, and cross sectional of the U‐PP with different thickness. e) Cycle tests and f) rate performances for U‐PP separators. Reproduced with permission.^[^ ^112^ ^]^ Copyright 2020, Royal Society of Chemistry.

Li et al. conducted a comparative analysis of the stacking patterns of four MOFs to evaluate their impact on separator performance (**Figure** **8**a).^[^ ^111^ ^]^ Among these MOFs, Y‐FTZB, characterized by a high packing density (Figure 8b), exhibited reduced interfacial impedance, increased response current, and superior cycling and rate capabilities. The findings indicate that pore size alone in MOFs does not solely inhibit polysulfides migration. The improved performance of Y‐FTZB can be credited to its more compact packing structure, which effectively impedes both polysulfides migration through the pore size and polysulfides diffusion through grain boundaries (Figure 8c). The MOF modification layer is affected not only by macroscopic stacking morphology, but also by the thickness of the modification layer. Han et al. synthesized UiO‐66 microparticles through a straightforward solvothermal reaction with an approximate size of 200 nm.^[^ ^112^ ^]^ These particles were then deposited onto a Celgard 2500 (PP) separator to create a composite separator. The resulting modified separators were labeled as U‐PP‐5, U‐PP‐3.5, U‐PP‐2, and U‐PP‐1, corresponding to different UiO‐66 modification layer thicknesses of 5, 3.5, 2, and 1 µm, respectively (Figure 8d). All these separators exhibited uniform thickness and dense accumulation, except for U‐PP‐1. Moreover, as the thickness of the UiO‐66 layer increased, its stacking arrangement became more compact. At 0.5 C, the capacity retention after 60 cycles improved from 87.57% to 95.75% as the thickness increased from 1 to 2 µm. However, it decreased from 95.75% to 81.89% as the modification thickness grew from 2 to 5 µm (Figure 8e). Remarkably, U‐PP‐2 displayed the best performance in cyclic voltammograms, long‐term cycling, and rate capability (Figure 8f), emphasizing the significance of the appropriate stacking morphology and thickness in facilitating Li^+^ transport, hindering polysulfide migration, and enhancing their absorption.

**Figure 9:**
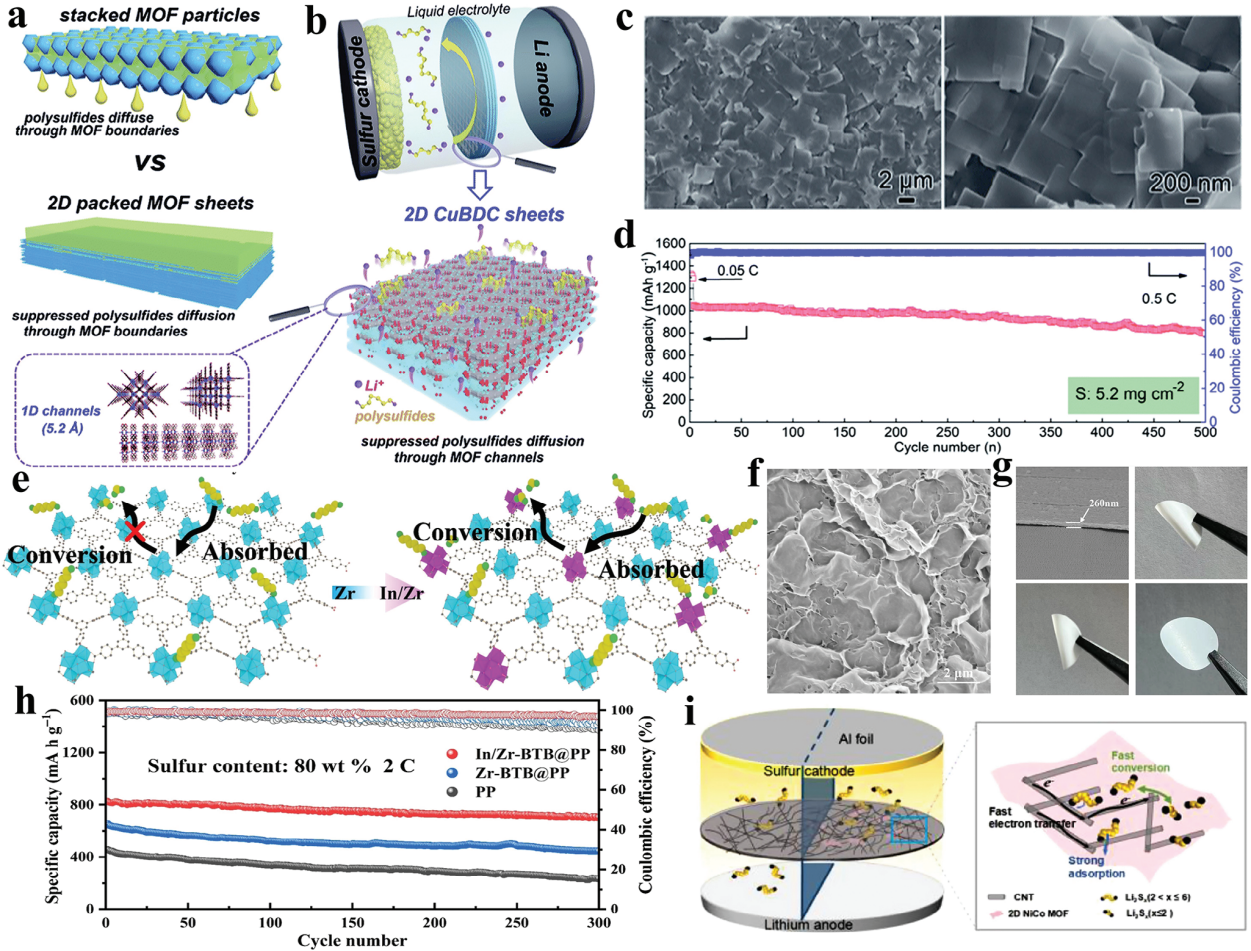
a) Diagram depicting the role of particle‐like MOFs and 2D MOF sheets in inhibiting the polysulfides diffusion. b) Illustration demonstrating the utilization of 2D CuBDC sheets as a functional separator in Li–S cells. c) SEM images of the CuBDC sheets. d) Cycling performance of the 2D‐CuBDC cell. Reproduced with permission.^[^ ^113^ ^]^ Copyright 2021, Royal Society of Chemistry. e) Schematic of the polysulfide absorption and conversion of In/Zr‐BTB and Zr‐BTB nanosheets. f) SEM image of In/Zr‐BTB nanosheet. g) SEM images of In/Zr‐BTB@PP separator. h) Cycling performance at 2 C. Reproduced with permission.^[^ ^114^ ^]^ Copyright 2023, Chinese Chemical Society. i) Schematic diagram of the 2D NiCo MOF/CNT interlayer in Li–S batteries. Reproduced with permission.^[^ ^115^ ^]^ Copyright 2023, Elsevier.

2D MOF nanosheets were promising modification layer for Li–S battery separators. Chang et al. proposed that 2D MOF nanosheets can be closely packed to create a boundary‐free layered structure, theoretically eliminating polysulfide shuttling through grain boundaries (**Figure** **9**a,b).^[^ ^113^ ^]^ They synthesized CuBDC sheets to be used as a modification layer for commercial separators. The SEM images illustrate that CuBDC exhibits a well‐defined nanosheet structure, with closely stacked layers (Figure 9c). The tightly stacked CuBDC sheets not only reduce the path for lithium‐ion transport but also effectively suppress the shuttle effect. They also offset the negative effects of increased polarization in the cycling process due to the small pore. Li–S batteries equipped with CuBDC as a functional modification layer achieved a satisfied capacity of 830 mAh g^−1^ after 1000 cycles of 1 C cycling at a high sulfur loading of 5.2 mg cm^−1^, demonstrating the excellent performance of 2D CuBDC sheets (Figure 9d). Cheng et al. achieved an ultrathin and lightweight modified separator In/Zr‐BTB@PP through an ion‐exchange method. The thickness and areal mass loading of the modified layer are as low as 260 nm and 0.011 mg cm^−2^, respectively (Figure 9e–g).^[^ ^114^ ^]^ The densely stacked In/Zr‐BTB nanosheets created a boundary‐free structure that effectively prevented the shuttle effect. The ultra‐thin modification layer reduces the Li^+^ transport distance, enabling rapid Li^+^ mobility. Simultaneously, the abundance of active indium sites enhances the adsorption of polysulfides and leading to efficient catalytic conversion. With the benefit of this innovative design, even when subjected to high sulfur content of 80 wt.% and cycled 300 times at 2 C, Li–S batteries employing the In/Zr‐BTB@PP separator maintained an impressive reversible capacity of 85.6%, demonstrating excellent cycle stability (Figure 9h and Table 1). Feng et al. also synthesized 2D ultrathin and ultralight bimetallic NiCo MOF nanosheets.^[^ ^115^ ^]^ These nanosheets feature a substantial specific surface area and an abundance of exposed catalytic sites, facilitating efficient adsorption and exhibiting robust catalytic activity (Figure 9i). Therefore, with a 2D NiCo MOF/CNT interlayer, the cell achieved an initial discharge capacity of 1132.7 mAh g^−1^ at 0.5 C, corresponding to 67.6% of the theoretical capacity of sulfur. Even after 300 cycles, it retained 709.1 mAh g^−1^, displaying excellent rate capability and cycling performance.

### 2.5 MOF Carbon Composites

In addition to the previously discussed design strategies for Li–S MOFs, MOF materials can also be combined with carbon material to create a free‐standing structure.^[^ ^116^ ^]^ This structure maintains MOFs' unique features while enhancing their conductivity, adsorption capacity, and ductility to withstand the demanding conditions within batteries.^[^ ^117^, ^118^ ^]^ Typical choices for combining MOFs include various conductive carbon‐based materials, like graphene and carbon nanotubes.

**Figure 10:**
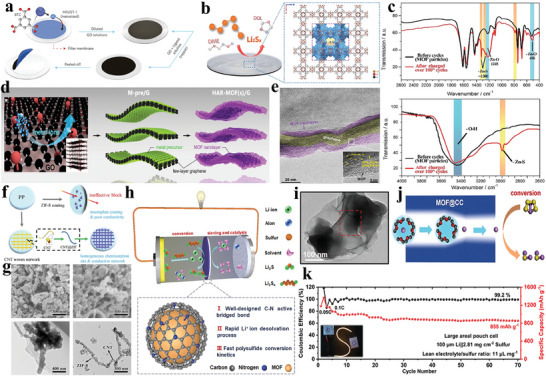
a) Illustration depicting the manufacturing process for producing MOF@GO separators. Reproduced with permission.^[^ ^121^ ^]^ Copyright 2016, Nature. b) Diagram illustrating the Zn‐based MOF@GO separator. c) The analyses conducted utilizing the IR spectra. Reproduced with permission.^[^ ^122^ ^]^ Copyright 2016, Royal Society of Chemistry. d) Diagram illustrating the preparation of MOF/graphene nanosheets. e) False‐color HRTEM image of ZIF‐67/G nanosheet. Reproduced with permission.^[^ ^123^ ^]^ Copyright 2020, American Chemical Society. f) Diagram depicting ZIF‐8 and the CNT@ZIF modified separators. g) SEM images and TEM images for CNT@ZIF‐45 and CNT@ZIF‐30. Reproduced with permission.^[^ ^124^ ^]^ Copyright 2018, Elsevier. h) Diagram showing the process of solvated Li^+^ migrating from the bulk solution to the surface of MOF@CC@PP. i) TEM image of the MOF@CC. j) Diagram depicting solvated Li^+^ ions within the channels of MOF. k) Cycle test of the large pouch cell using MOF@CC@PP. Reproduced with permission.^[^ ^125^ ^]^ Copyright 2023, Wiley‐VCH.

Graphene, along with its derivatives graphene oxide (GO) and reduced graphene oxide (rGO), falls under the category of 2D carbon materials comprising sp^2^ hybridized carbon atoms.^[^ ^119^, ^120^ ^]^ These materials offer a superior electrical conductivity and high specific surface area. Bai et al. selected HKUST‐1, a MOF with highly ordered microporous windows, to create composite separators in combination with GO (**Figure** **10**a).^[^ ^121^ ^]^ The MOF grown in situ on the filter membrane has micropores much smaller than the diameter of polysulfides, thus effectively obstructing polysulfides while selectively permitting the passage of lithium ions. The GO layer, which covers the MOF, functions as both a barrier and an enhancer for the stability of the separator by filling gaps in the MOF interlayer. Li–S batteries equipped with the HKUST‐1@GO composite separator preserve a discharge capacity of 855 mAh g^−1^ even after 1500 cycles, demonstrating exceptional cycling stability. Bai et al. similarly employed a Zn‐based MOF (Zn‐HKUST‐1) to obtained a composite separator with GO through the same process (Figure 10b).^[^ ^122^ ^]^ Infrared spectroscopy revealed the presence of Zn‐S bonds formed during charging and discharging, contributing to the MOF structure's stability (Figure 10c). The separator based on Zn‐HKUST‐1@GO exhibited a solid cycling performance, retaining a capacity of 657 mAh g^−1^ after 1000 cycles at 1 C. Beyond the composite of GO and MOF, the enrichment of functional groups on GO also opens up the possibility of in situ growth of MOF on GO. Liu et al. employed ZIF‐67 composite graphene to prepare a high‐aspect‐ratio MOF/graphene composite material (HAR‐MOF/G), which was loaded onto a PP membrane, obtained a high‐performance Janus ion sieve membrane structure (Figure 10d).^[^ ^123^ ^]^ This HAR‐MOF/G material addressed issues related to grain boundaries in conventional particulate MOFs and enhanced the limited electrical conductivity of MOFs (Figure 10e). These improvements prevented the depletion of sulfur species and enhanced the overall performance. When applied in Li–S cells, the Janus membrane demonstrated remarkable stability during extended cycling, maintaining 75.3% of its capacity after 1700 cycles at 2 C, revealing outstanding rate capability and cycling stability.

CNTs are 1D nanomaterials known for their distinct mechanical, conductive, and chemical properties. Combining CNTs with MOFs in composites not only enhances the film‐forming characteristics of MOFs but also augments their conductivity.^[^ ^34^ ^]^ Wu et al. devised a CNT@ZIF composite and employed it to fabricate a functionalized separator aimed at sequestering dissolved polysulfides via Lewis acid‐base interactions between the polysulfides and ZIF‐8 framework (Figure 10f).^[^ ^124^ ^]^ As depicted in Figure 10g, the surface of MWCNTs is densely covered with numerous ZIF‐8 nanocrystals. With the enhanced conductivity and the synergistic effect facilitated by CNT, the functionalized separator can efficiently restrict polysulfides shuttling, capturing and recycling them. The Li–S cell utilizing the CNT@ZIF functionalized separator demonstrates remarkable improvements in reversible capacity and cycle performance, achieving an impressive initial discharge capacity of 1588.4 mAh g^−1^ at a 0.2 C, corresponding to 94.8% of the theoretical capacity of sulfur. Furthermore, it shows an enhanced capacity retention rate of 36.2% after one hundred cycles compared to the battery without the functional layer. Combining MOF materials with carbon materials or modifying MOFs on carbon hosts has significantly enhanced the conductivity and film‐forming properties of MOFs, resulting in improved catalytic activity. However, there is limited research on coating carbon materials onto MOF surfaces for catalyzing the conversion of polysulfides. Herein, Li et al. produced catalytic composites (MOF@CC) by connecting NH_2_‐UiO‐66 with composite carbon and coated them onto a polypropylene separator (MOF@CC@PP) (Figure 10h).^[^ ^125^ ^]^ This setup encourages the transformation of Li^+^ solvation structure turns into anion‐involved mode, resulting in accelerated Li^+^ diffusion and prevention the accumulation of polysulfides. Simultaneously, the highly conductive carbon layer tightly interfaces with the MOF particles, providing a pathway for electron exchange during the redox transformation of sulfur species (Figure 10i). Dissolved polysulfides in the electrolyte are captured by MOF@CC and interact with bare Li^+^ from the Li^+^‐solvent complex. Additionally, the close connection between MOF and carbon can also facilitate the reaction between polysulfides and exposed Li^+^ (Figure 10j). A Li–S cell equipped with the bifunctional MOF@CC@PP exhibits a capacity of 765 mAh g^−1^ at 5 C, and the large pouch cell maintains a stable discharge capacity of 855 mAh g^−1^ after 70 cycles (Figure 10k). This demonstrates excellent reaction kinetics and rate performance.

### 2.6 MOF Polymer Composites

Functional polymer materials boast a higher number of functional groups in comparison to conventional PP separators. The pliability and ease of processing of polymer materials simplify the preparation of MOF‐polymer composite separators.^[^ ^126^ ^]^ Furthermore, the broader range of material sources for polymers, compared to carbon materials, enhances the adaptability and diversity of modification strategies.^[^ ^127^ ^]^

**Figure 11:**
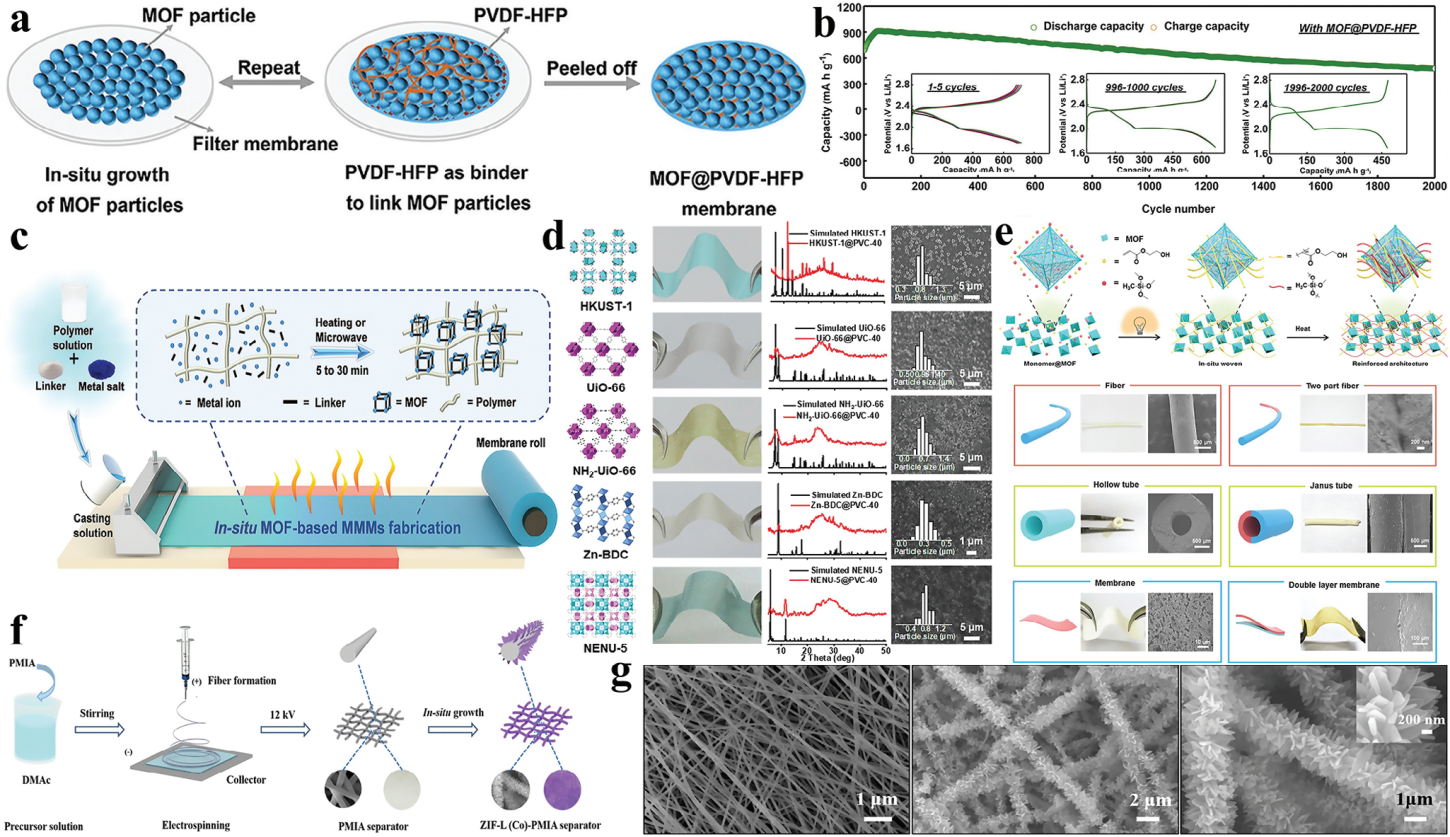
a) Illustration depicting the MOF@PVDF‐HFP membrane structure. b) Ultralong cycling performance of MOF@PVDF‐HFP cell at 2 C. Reproduced with permission.^[^ ^128^ ^]^ Copyright 2018, Wiley‐VCH. c) Schematic representation of the in situ heat‐assisted solvent‐evaporation method used for creating MOF‐based MMMs. d) Illustration depicting the structure, SEM images, and PXRD patterns of the MMMs. Reproduced with permission.^[^ ^129^ ^]^ Copyright 2020, Wiley‐VCH. e) Characterization results of various MOF‐based devices fabricated using the two‐step PHAP method. Reproduced with permission.^[^ ^130^ ^]^ Copyright 2021, Wiley‐VCH. f) Diagram outlining the fabrication process of the Z‐PMIA separator. g) SEM images of PMIA and Z‐PMIA separators. Reproduced with permission.^[^ ^133^ ^]^ Copyright 2021, Elsevier.

He et al. utilized Poly(vinylidenefluoride‐co‐hexafluoropropylene) (PVDF‐HFP) as a binding agent, laminating it with HKUST‐1 to create a flexible MOF@PVDF‐HFP separator (**Figure** **11**a).^[^ ^128^ ^]^ MOF membranes tightly integrated with polymers effectively utilize the narrow MOF pores to inhibit polysulfide shuttling, ensuring uniform Li^+^ flow, inhibiting Li dendrite growth, and enabling a uniform Li plating and peeling process. Therefore, Li–S batteries exhibit extended cycle life, with 2000 cycles at a 2 C current and an average degradation rate per cycle of 0.015% (Figure 11b). Beyond polymer's role in binding the MOF, the molding method employed for the MOF film is also intricately linked to its performance. Gao et al. described an in situ heat‐assisted solvent‐evaporation method (Figure 11c) to conveniently manufacture MMM based on MOFs, which are characterized by uniformity, stability, loading, and thickness (Figure 11d).^[^ ^129^ ^]^ This separator exhibits an impressive specific capacity of 1163.7 mAh g^−1^ and maintains a low average decay rate of 0.08% per cycle over 700 cycles at 0.5 C, showcasing its relatively superior performance. They also introduced a two‐step photoinduced heat‐assisted processing (PHAP) method that leverages the benefits of photoinitiated polymers and MOFs to create a triple‐layer separator based on MOFs with stepped channels (Figure 11e).^[^ ^130^ ^]^ This triple‐layer MMM combines the advantages of small pore size to suppress polysulfides and large pore size to facilitate Li^+^/electrolyte transfer. It effectively overcomes the cell polarization attributed to having two distinct pore sizes in the two‐layer MOF‐based MMM. The MOF‐based triple‐layer separator exhibited a decent specific capacity of 1365.0 mAh g^−1^ and demonstrated a more favorable cycling performance with a 0.03% capacity fade per cycle from the 100th to the 700th cycle, exhibiting the outstanding promise of MOF‐based MMM in Li–S batteries. The electrospinning technology is a greatly effective method to prepare nanofiber separators with special structures for Li–S cells owing to its high specific surface area and interconnected pores.^[^ ^131^, ^132^ ^]^ Liu et al. employed this method to create aramid nanofiber separators (Z‐PMIA separators) (Figure 11f).^[^ ^133^ ^]^ These separators were fabricated by growing ZIF‐L(Co) directly on electrospun PMIA nanofibers. The resultant separators displayed a uniform distribution of pore sizes and enhanced mechanical resilience. This unique structure supported uniform nucleation and growth of Li anodes. The blade‐like nanostructured ZIF‐L(Co) coating on the fibers (Figure 11g) not only improved the rapid transfer of Li ions and electrolyte diffusion but also provided a substantial surface area featuring with an abundance of active metal sites, which are beneficial for polysulfides adsorption and conversion. Consequently, the Z‐PMIA separator demonstrates a remarkable initial discharge capacity of 1391.2 mAh g^−1^, corresponding to 83% of the theoretical capacity of sulfur. Even after 350 cycles at 0.2 C, it maintains a discharge capacity of 961.1 mAh g^−1^, with a minimal capacity decrease of only 0.033% per cycle. Furthermore, the well thermal stability and mechanical strength of PMIA allowed the Z‐PMIA separators to maintain high charge/discharge performance even at elevated temperatures of 80 °C.

## 3 Conclusion and Outlook

Li–S battery, with its high energy density and theoretical discharge capacity, stands as a highly sought‐after energy storage technology. The utilization of MOF materials to modify Li–S battery separators has achieved substantial attention from researchers in recent years. Nonetheless, challenges such as the notorious shuttling effects and low sulfur utilization require modified separators that can effectively mitigate these issues and expedite polysulfides conversion. Fortunately, the inherent structural adaptability of MOF materials has paved the way for innovative design strategies, resulting in significant advancements in separator modification. These developments hold the promise of creating multifunctional modified separators that address mechanical properties, polarization effects, and the inhibition of the shuttle effect. While the progress made in MOFs for Li–S batteries is encouraging, there are still challenges that require attention and resolution. The predominant issue lies in the inferior Li^+^ conductivity of MOF‐based modification layers, exacerbated by the often‐excessive thickness of these layers, which can offset the advantages of using MOFs in separator modification. To fully leverage the distinctive advantages of MOFs in Li–S batteries, novel concepts and methodologies should be introduced. The following perspectives shed light on potential avenues for further exploration.

1. Efforts can be made to utilize single crystal technology to decipher the adsorption/conduction sites of MOFs for polysulfides, with the potential to achieve precise identification of adsorption/conduction sites for polysulfides. This approach allows for a systematic exploration of the ion‐selective conduction of MOFs and their effects on polysulfides.
2. The interparticle spaces within MOFs may still allow the passage of polysulfides. Simultaneously, the internal resistance of the cell grows with coating thickness, potentially affecting overall cell performance. This suggests that choosing a thin and compact modified layer comprised of 2D porous MOF materials on the separator would be a more advantageous option.
3. It is highly desirable to rationally design a multifunctional MOF with simultaneous high efficiency in intercepting polysulfides and promoting rapid Li^+^ transport. Currently, MOF‐based materials used for separator modification primarily include star MOFs such as ZIF‐8, ZIF‐67, UIO‐66, and their composites. Exploring new multifunctional MOF materials can be attempted by synthesizing novel MOFs using imidazole, triazole, nitroimidazole ligands, and functional pore‐space‐partition agents. Incorporating imidazole ligands into the parent MOF framework or utilizing pore‐space‐partition agents not only effectively enhances the material's stability but also provides tunable Lewis acid sites. This approach may induce electrostatic interactions between MOFs and polysulfides, enhancing the interception capability of MOFs toward polysulfides.
4. Efforts should be made to ensure that the constructed MOF‐modified layer exhibits excellent Li^+^ transport capabilities and catalyzes the conversion of intercepted polysulfides. This is essential to ensure stable, long‐cycle operation of high‐sulfur‐loading electrodes under high current rate conditions. Considerations may include tuning MOF ligands and adjusting pore sizes for selectively and efficiently transmitting Li^+^. Additionally, the selection of central metals such as Co, Ni with outstanding polysulfides catalytic capabilities is crucial. This approach not only enhances Li^+^ transport but also accelerates the efficient catalytic conversion and reutilization of intercepted polysulfides.

In light of the aforementioned discussions, it is evident that innovative ideas and materials based on MOFs offer significant potential for propelling the progress of high‐performance Li–S batteries. With the growing focus of researchers in this domain, fewer challenges will be encountered, leading to an upsurge in breakthroughs and the continued expansion of the potential of MOF materials.

## Conflict of Interest

The authors declare no conflict of interest.
